# Evaluation of Clinical Performance of Moisture-tolerant Sealant on Pit and Fissure Caries in Two Different Setting Conditions: A Randomized Controlled Trial

**DOI:** 10.1055/s-0045-1814727

**Published:** 2026-01-22

**Authors:** Shweta Dangi, Kumar Gaurav Chhabra, Naganandini S., Pankaj Chaudhary, Seemadevi T., Harshit Srivastava

**Affiliations:** 1 Nims Dental College and Hospital, Nims University Rajasthan, Jaipur, India; 2 Research and Development Cell, Datta Meghe Institute of Higher Education and Research, Wardha, Maharashtra, India

**Keywords:** dental care for children, dental caries, Embrace WetBond, pit and fissure sealants

## Abstract

**Objective:**

The moisture-tolerant resin-based sealant Embrace WetBond (EWB), designed for effective bonding in moist conditions, offers a promising solution for caries prevention in challenging clinical environments. This study aimed to evaluate and compare its retention rate and caries-preventive efficacy in newly erupted permanent molars under two distinct clinical settings.

**Materials and Methods:**

This split-mouth randomized controlled trial assessed EWB sealant under moisture-controlled and non-moisture-controlled conditions in schoolchildren aged 6 to 14 years with bilateral, non-cavitated molars. A total of 37 participants (112 teeth; 56 per group) were randomized, each serving as their own control. Sealant retention, caries prevention, marginal discoloration, marginal adaptation, color match, and smoothness of surface were assessed at 1, 6, and 12 months using modified USPHS criteria. Statistical analysis included the chi-square test, Mann-Whitney U test, and Friedman test to compare retention rates and sealant performance over time.

**Results:**

A total of 37 children (56 teeth per group) were included, with balanced baseline characteristics due to the split-mouth design. At 12 months, sealant retention significantly declined in both groups (Group I: 96.4% at 1 month to 49% at 12 months; Group II: 96.4 to 54.9%; *p* < 0.001), though no significant differences were observed between groups ( *p* > 0.05). Caries incidence was minimal, with only 2% of teeth affected at 12 months in each group. Apart from retention, no other parameters showed significant deterioration over 12 months.

**Conclusion:**

Pit and fissure sealants demonstrated similar effectiveness in preventing occlusal caries under both moisture-controlled and non-moisture-controlled conditions over 12 months. Their comparable performance in limited moisture-controlled settings highlights their suitability for real-world clinical practice and public health programs.

## Introduction

Dental caries develops when bacterial biofilms exposed to fermentable carbohydrates disrupt the demineralization–remineralization balance, and because pits and fissures on permanent molars trap debris and bacteria; these surfaces—though constituting only 12.5% of the dentition—are responsible for more than 50% of caries in school-aged children. 1 2 3 4 Dental caries is eight times more likely to occur in pits and fissures than on a smooth surface. 5 Because the average tooth brush bristle (0.2 mm) is too big to enter most fissures, the morphology makes mechanical debridement methods inaccessible, increasing the susceptibility of fissures to caries by a factor of many. 6 Early application of occlusal sealants on first permanent molars is crucial in high-risk children, as newly erupted, less mineralized teeth are highly vulnerable to caries. 7 8

Pit and fissure sealants act by creating a micromechanical bond with the enamel surface, thereby blocking nutrient access to caries-causing bacteria, and their long-term therapeutic success is strongly influenced by factors such as the morphology of pits and fissures, the adequacy of isolation during placement, and the quality of enamel conditioning. 9 10 The main disadvantage of resin-based sealants is their susceptibility to moisture, which eventually causes the sealant to fail in terms of retention. 11 Consequently, the material of choice would be a sealer with enhanced physical qualities, strong moisture tolerance, and good retention capabilities. 12

Embrace WetBond (EWB) is a moisture-tolerant, Bis-GMA-free resin sealant that maintains strong retention by balancing hydrophilic and hydrophobic properties. 13 Children who have trouble controlling their wetness can apply this sealant more easily since it is less technique sensitive and easy to handle. 14

There are many studies on the retention and caries-preventive effects of hydrophilic sealants. In the majority of studies, it is proven to be efficacious over the hydrophobic resin-based sealants and glass ionomer-based sealants. 13 15 16 17 18 Considering its beneficial aspects, it can be used in outreach programs. Hence, this study was conducted to check and compare the retention and caries-preventive effects of EWB in two different settings in young permanent molars, i.e., moisture-controlled and non-moisture-controlled.

## Materials and Methods

The study was performed in accordance with Good Clinical Practice guidelines and conformed to the Declaration of Helsinki. Ethical approval was obtained from the Institutional Ethical Committee before the conduct of the study (IEC Number: IEC/P-260/2023). The study protocol has been registered at the Clinical Trials Registry – India (CTRI) (Trial Registration Number: CTRI/2024/06/068418).

This is an interventional, parallel design, split-mouth, prospective randomized controlled trial to assess and compare the clinical performance of moisture-tolerant pit and fissure sealant in a moisture-controlled setting and a non-moisture-controlled setting. The moisture-tolerant pit and fissure sealant is the EWB (Pulpdent Corporation, MA, USA). The inclusion criteria for the study are:

1. Questionable, non-cavitated, bilateral pits and fissures in permanent maxillary or mandibular first molars in children ranging from 6 to 10 years of age.
2. Questionable, non-cavitated, bilateral pits and fissures in permanent maxillary or mandibular second molars in children ranging from 11 to 14 years of age.
3. Children who are willing to attend the scheduled follow-ups.

The exclusion criteria for the study are:

1. Children with enamel defects such as amelogenesis imperfecta, enamel hypoplasia, and dental fluorosis.
2. The occlusal surface of molars having shallow pits and fissures, which are self-cleansing in the oral cavity for more than 4 years.
3. Molars with caries in the proximal surfaces.
4. Previously placed sealants or restorations on molar teeth.
5. Uncooperative children.

Although the study focused on children within defined age groups and specific occlusal conditions, formal caries risk assessment measures such as past caries experience, plaque index, or ICDAS-based risk classification were not applied in the inclusion criteria.

The required sample size was determined to be 100 teeth using G*Power software version 3.1.9.4, with a study power (β) of 80%, an effect size of 0.51, degrees of freedom set at 5, and an α (α) error of 5%. The primary outcome variable considered for the sample size calculation was the retention rate and presence of caries, based on reference data from the Priyadharshini et al's study, which informed the estimated effect size of 0.51. These assumptions were used to ensure adequate statistical power for detecting clinically relevant differences. 8 The sample size derived was 50 teeth in each group. Taking into account a 10% attrition rate, 112 teeth were selected and sealed. The study was conducted in three schools of Amer Block, Jaipur, Rajasthan, from June 2024 to June 2025. Two schools were selected randomly through a lottery method. Prior permission was obtained from the concerned school authorities before the examination of the children. A total of 240 children were screened in natural daylight. Two examination methods were used for the clinical examination of molars: visual and tactile. For the visual examination, cleaning and drying of the tooth were done manually with the help of a chip blower, and for the tactile examination, a round-tip dental probe was used to clean the plaque and any food debris present on the pits and fissures. Of these 102 children who met the selection criteria, consent forms explaining the need for the pit and fissure sealants were given to the children's parents or guardians who fulfilled the inclusion criteria. Consent was obtained to participate in the study. Among them, only 37 children brought back the duly signed consent forms, which were then finally recruited in the study. The complete participant recruitment flowchart as per CONSORT guidelines is shown in Fig. 1 .

**Fig. 1:**
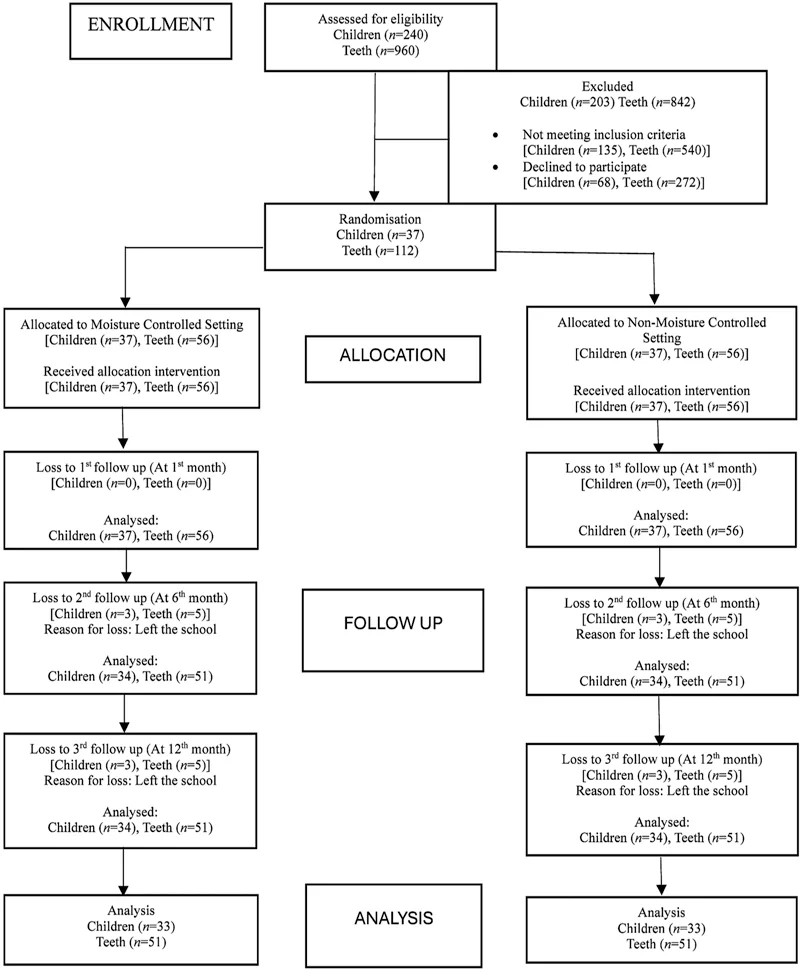
CONSORT flowchart depicting the participant recruitment and follow-up.

The entire study consisted of a total of five appointments per child. In each child, the coin toss method was used to select the side for a non-moisture-controlled setting. The sealant was first applied under a non-moisture-controlled setting on the first day, and later on the subsequent day, the sealant was applied under a moisture-controlled setting at the nearest dental outreach center, and approval was taken from the school authorities and parents for the same using the consent forms. Randomization was done by a third person who was not aware of the study. The assessor was blinded to the sequencing and allocation of the groups. A single calibrated investigator performed all the procedures of pit and fissure sealant application. Intra-examiner reliability for clinical evaluation using the USPHS/Ryge criteria was calculated on a separate group of children prior to the start of the study, yielding a Cohen's Kappa value of 0.76, which indicated substantial agreement. The application of the sealant was done according to the manufacturer's instructions. Although the assessor was blinded to group allocation, participant (child) blinding was not feasible as the interventions were performed in different settings on separate days.

Non-moisture-controlled setting: For the sealant application, the steps were: tooth cleaning with cotton pellets and a dental probe, rinsing with water, air drying with a chip blower and cotton pellets, application of phosphoric gel (38%) for 15 seconds, rinsing with water vigorously for 30 seconds and air drying and keeping the occlusal surface as slightly moist, shiny, or glossy, sealant application using applicator tip no. 22 attached to a syringe at an angle to the cuspal walls, light curing of the sealant for 20 seconds using visible light cure unit, and evaluation of sealant for complete coverage, voids or bubbles, occlusion, and marginal adaptation.

Moisture-controlled setting: For the sealant application, the steps were similar except that the air drying was done with the help of a dental unit air–water spray, and proper isolation was maintained with the help of preformed cotton rolls.

Evaluation of the retention, caries protective effect, marginal integrity, marginal discoloration, color match, and surface smoothness of these sealants was clinically done using the modified Ryge criteria/the United States Public Health Service (USPHS) for Direct Clinical Evaluation of Restorations criteria. The sealants were evaluated at the end of the 1st, 6th, and 12th months from the date of placement as depicted in Fig. 2 . The outcomes were assessed by an investigator who was blinded to the interventional group.

**Fig. 2:**
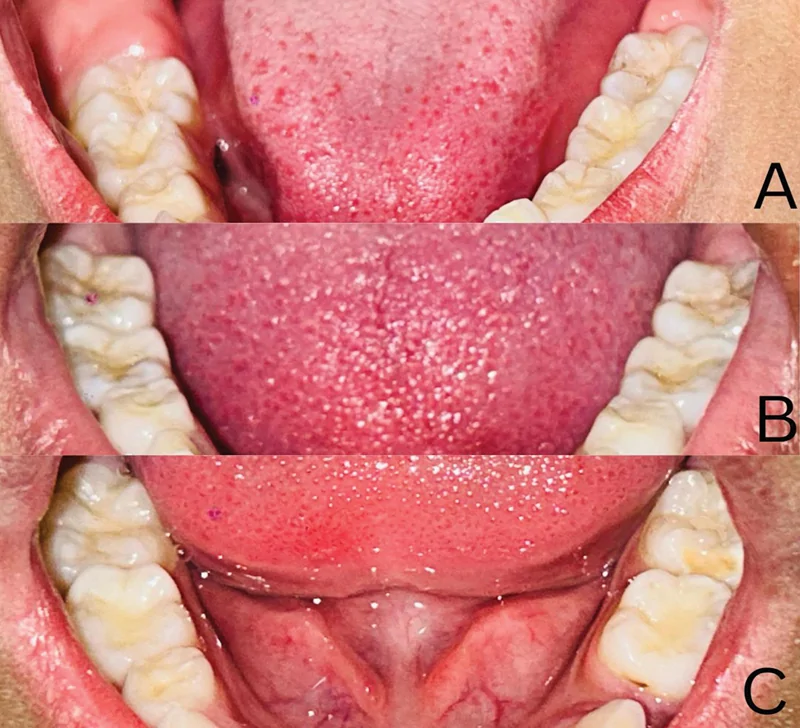
Sealant evaluation at ( **A** ) 1st, ( **B** ) 6th, and ( **C** ) 12th month follow-up.

The data were collected and entered in MS Excel, and then statistical analysis was performed using SPSS version 26. The normality of the data was assessed using the Kolmogorov-Smirnov test and the Shapiro-Wilk test, and the data were found not to be normally distributed. The comparisons for all the outcomes were made using Mann-Whitney U tests for the intergroup comparisons between moisture-controlled and non-moisture-controlled setting groups. The outcomes comparison among 1st, 6th, and 12th months was done using the Friedman test for intragroup comparisons.

This comprehensive methodology adheres to the CONSORT guidelines (2010), ensuring transparency, reproducibility, and robustness in the study's execution and reporting.

## Results

There were a total of 37 children overall, with 56 teeth in each group. Among the school children, 37.5% were female and 62.5% were male, with an average age of 10.73 ± 0.94 years. Due to the split-mouth design of the study, the demographics of both groups were the same. Mandibular first molars accounted for 62.5% of the sealed teeth, followed by maxillary first molars (33.9%) and mandibular second molars (3.6%).

The clinical parameters for Group I (moisture-controlled) restorations assessed at the 1-, 6-, and 12-month marks are shown in Table 1 . The percentage of fully retained restorations decreased from 96.4% at 1 month to 66.7% at 6 months and 49% at 12 months ( *p* < 0.001), indicating a drop in retention rates with time. Only one instance (2%) of caries development was seen at the 12-month mark ( *p* = 0.006). At 12 months, 90.2% of restorations had no discoloration, but 7.8% had superficial staining and 2% had deep staining ( *p* < 0.001). This indicates that marginal discoloration increased with time. At every time point, marginal adaptation was at its best ( *p* = 0.007). At 12 months, color match drastically declined, with 78.4% displaying no mismatch, 17.6% displaying noticeable but manageable mismatch, and 4% having clinically poor aesthetics ( *p* < 0.001). About 98% of restorations maintained surface smoothness comparable to neighboring tooth structure after 12 months, indicating only little changes in surface smoothness ( *p* = 0.006). According to these results, memory and aesthetic qualities gradually decreased with time, but functional characteristics like smoothness and marginal adaptability were mostly constant.

**Table 1:** Clinical parameters for Group I (moisture-controlled) at 1st, 6th, and 12th month

Clinical parameter		At 1st month ( *n* = 56)	At 6th month ( *n* = 51)	At 12th month ( *n* = 51)	*P* value (Friedman test)
Retention	A: Retained	54 (96.4%)	34 (66.7%)	25 (49%)	<0.001*
	B: Partially retained	2 (3.6%)	17 (33.3%)	10 (19.6%)	
	C: Missing	0 (0%)	0 (0%)	16 (31.4%)	
Caries formation	A: No evidence of caries	56 (100%)	51 (100%)	50 (98%)	0.006*
	B: Evidence of caries along the margin of the restoration	0 (0%)	0 (0%)	1 (2%)	
Marginal discoloration	A: No discoloration anywhere along the margin	56 (100%)	51 (100%)	46 (90.2%)	<0.001*
	B: Superficial staining (removable, usually localized)	0 (0%)	0 (0%)	4 (7.8%)	
	C: Deep staining	0 (0%)	0 (0%)	1 (2%)	
Marginal adaptation	A: Undetectable crevice along the margin	56 (100%)	51 (100%)	51 (100%)	0.007*
	B: Detectable V-shaped defect in enamel only	0 (0%)	0 (0%)	0 (0%)	
	C: Detectable V-shaped defect in DEJ	0 (0%)	0 (0%)	0 (0%)	
Color match	A: No shade mismatch in room light in 3 to 4 seconds	56 (100%)	50 (98%)	40 (78.4%)	<0.001*
	B: Perceptible mismatch but clinically acceptable	0 (0%)	1 (2%)	9 (17.6%)	
	C: Aesthetically unacceptable (clinically unacceptable)	0 (0%)	0 (0%)	2 (4%)	
Smoothness of surface	A: Restoration is as smooth as the adjacent tooth structure	56 (100%)	51 (100%)	50 (98%)	0.006*
	B: Restoration is rougher than the adjacent tooth structure	0 (0%)	0 (0%)	1 (2%)	
	C: Restoration is rougher than the adjacent tooth structure and contains pit and fissures	0 (0%)	0 (0%)	0 (0%)	

Table 2 represents the observed clinical parameters in Group II and the statistical test employed is Friedman test. In the non-moisture-controlled group, sealant retention significantly declined over time, with 96.4% of sealants fully retained at 1 month, dropping to 62.7% at 6 months, and further to 54.9% at 12 months ( *p* < 0.001). Caries formation was minimal, with no evidence of caries at 1 and 6 months, and a slight increase to 2% at 12 months ( *p* = 0.006). Marginal discoloration increased over time, with no discoloration at 1 month, 2% at 6 months, and 13.7% at 12 months ( *p* < 0.001). Marginal adaptation remained consistently high at 100% throughout the study period ( *p* = 0.007). Color match showed a significant decline, with 100% at 1 month, 98% at 6 months, and 78.4% at 12 months ( *p* < 0.001). Surface smoothness remained high, with 100% at 1 and 6 months, and a slight decrease to 98% at 12 months ( *p* = 0.006).

**Table 2:** Clinical parameters for group II (non-moisture controlled) at 1st, 6th, and 12th month

Clinical parameter		At 1st month ( *n* = 56)	At 6th month ( *n* = 51)	At 12th month ( *n* = 51)	*P* value (Friedman test)
Retention	A: Retained	54 (96.4%)	32 (62.7%)	28 (54.9%)	<0.001*
	B: Partially retained	2 (3.6%)	19 (37.3%)	7 (13.7%)	
	C: Missing	0 (0%)	0 (0%)	16 (31.4%)	
Caries formation	A: No evidence of caries	56 (100%)	51 (100%)	50 (98%)	0.006*
	B: Evidence of caries along the margin of the restoration	0 (0%)	0 (0%)	1 (2%)	
Marginal discoloration	A: No discoloration anywhere along the margin	56 (100%)	50 (98%)	44 (86.3%)	<0.001*
	B: Superficial staining (removable, usually localized)	0 (0%)	1 (2%)	6 (11.7%)	
	C: Deep staining	0 (0%)	0 (0%)	1 (2%)	
Marginal adaptation	A: Undetectable crevice along the margin	56 (100%)	51 (100%)	51 (100%)	0.007*
	B: Detectable V-shaped defect in enamel only	0 (0%)	0 (0%)	0 (0%)	
	C: Detectable V-shaped defect in DEJ	0 (0%)	0 (0%)	0 (0%)	
Color match	A: No shade mismatch in room light in 3 to 4 seconds	56 (100%)	50 (98%)	40 (78.4%)	<0.001*
	B: Perceptible mismatch but clinically acceptable	0 (0%)	1 (2%)	10 (19.6%)	
	C: Aesthetically unacceptable (clinically unacceptable)	0 (0%)	0 (0%)	1 (2%)	
Smoothness of surface	A: Restoration is as smooth as the adjacent tooth structure	56 (100%)	51 (100%)	50 (98%)	0.006*
	B: Restoration is rougher than the adjacent tooth structure	0 (0%)	0 (0%)	1 (2%)	
	C: Restoration is rougher than the adjacent tooth structure and contains pit and fissures	0 (0%)	0 (0%)	0 (0%)	

At the 1st month follow-up, both groups exhibited excellent and comparable clinical outcomes, with no statistically significant differences observed across any of the evaluated parameters ( *p* = 1.000 for all). At the 6th month follow-up, both groups continued to show favorable clinical outcomes, with no statistically significant differences between the groups in any of the evaluated parameters ( *p* > 0.05). At the 12-month follow-up, both groups demonstrated comparable clinical performance, with no statistically significant differences observed across any parameters ( *p* > 0.05).

The Friedman test was used to perform pairwise comparisons of clinical parameters at three time intervals—1, 6, and 12 months—for both groups. A statistically significant change in retention was observed over time in both groups, specifically between 1 and 6 months ( *p* = 0.032 for Group I; *p* = 0.021 for Group II), and between 1 and 12 months ( *p* < 0.001 for both groups). However, changes in retention between 6 and 12 months were not significant in either group. For all other clinical parameters—caries formation, marginal discoloration, marginal adaptation, color match, and smoothness of surface—no statistically significant differences were found across the time intervals ( *p* > 0.05). This indicates that apart from retention, other clinical parameters remained stable over the 12-month evaluation period in both groups.

## Discussion

Pit and fissure sealants play a crucial role in preventive dentistry by protecting plaque-retentive grooves on occlusal surfaces that are highly prone to caries. 19 Pit and fissure sealants offer a non-invasive and effective method to prevent or halt occlusal caries. 20 The effectiveness of sealants in preventing caries depends on their secure bonding and complete retention, which in turn requires meticulous cleaning and drying of the deep and uneven pits and fissures. 21 Resin-based sealants are widely used currently, but their success relies on precise application, good patient cooperation, and ensuring a moisture-free environment during placement. 22 A significant disadvantage of traditional resin sealants is their hydrophobic nature, making them highly susceptible to moisture contamination. 23 Due to their moisture and technique sensitivity, hydrophobic resin sealants are unsuitable for field use, whereas EWB sealants offer a moisture-tolerant, hydrophilic alternative designed to address these limitations. 24 This newly developed material is moisture-tolerant, forms micromechanical and chemical bonds on damp surfaces, and activates in moisture, making it suitable for slightly moist teeth. 25 EWB is the first hydrophilic sealant with a Resin Acid-Integrated Network (R.A.I.N.), designed for moisture tolerance, fluoride release, pH-controlled activation, and dual chemical and micromechanical bonding. 26

The study used a split-mouth design, applying EWB under moisture-controlled and non-moisture-controlled conditions on opposite sides of the same patient to minimize individual variability in factors like oral hygiene, diet, and behavior. In this study, pits and fissures were cleaned using an explorer and water rinse—a method shown by Donnan and Ball to achieve 97% retention, proving as effective as cleaning with a handpiece and pumice. 27 In this study, 37% phosphoric acid was used for 20 seconds as per guidelines to create micromechanical enamel tags for sealant bonding, with prior trials showing no significant difference in retention whether adhesive was used or not. 28 29 Consequently, in the present study, sealants were placed directly onto etched surfaces without the application of an adhesive. Sealant polymerization was standardized in both groups using a single LED light-curing unit (>300 mW/cm ^2^ ) for 20 seconds, following manufacturer guidelines to ensure consistent retention outcomes.

Moreover, mandibular first permanent molars were selected in the majority of studies to evaluate the retention of pit and fissure sealants, as they offer greater visibility compared with other teeth. 30 In this study, only teeth within 4 years of eruption were included, based on findings from a systematic review by Azarpazhooh and Main, 31 which suggested that sealing teeth within this period is beneficial. Newly erupted teeth are highly prone to occlusal caries due to porous, debris-filled fissures, but their honeycombed enamel structure enhances sealant interlocking, lowering caries risk and effects of sealant loss. 21 32 Sealants were applied soon after tooth eruption, the optimal time recommended by literature, as teeth are most vulnerable to caries while root development and mineralization continue post-eruption. 33 34

At present, there is no standardized method for assessing and reporting the adequacy of sealed surfaces, creating challenges for comparative analysis and evaluation during recall visits. In this study, the examination method used was a modified version of the USPHS and Ryge's criteria, which assesses sealant retention, caries formation, marginal discoloration, marginal adaptation, color match, and surface smoothness.

Sealant loss occurs in two phases—early loss from application errors and later loss from occlusal wear—with most failures in the first 6 months linked to poor technique or tooth selection. 35 Given the highest likelihood of sealant failure shortly after placement, clinical evaluation for partial or total loss should be conducted within 1 year, with regular assessments to ensure complete retention.

The findings of the present study demonstrated no significant difference in retention or caries development between moisture-controlled setting and non-moisture-controlled setting. This suggests that moisture-tolerant resin-based sealants may serve as a valuable advancement in pit and fissure sealants.

Retention rates showed a gradual decline over time in both groups. At the 1-month follow-up, sealant retention was excellent, with 96.4% of restorations fully retained in both groups. At the 1-month follow-up, retention rates were higher in our study compared with three other studies. 5 21 36 However, at 6 months, retention had reduced to 66.7% in Group I and 62.7% in Group II, and at 12 months, it further dropped to 49 and 54.9%, respectively. At the 6th month the retention rates are seen to be comparable in one study 36 while most studies 5 8 11 13 14 15 16 17 18 37 38 39 40 show higher retention rates than our study and only three studies 21 24 25 show contrasting results which show lesser retention rates. At the 12th month, the retention rates were seen to be higher than our study in majority of the studies. 5 11 13 14 15 16 17 18 40 However, few studies 12 21 24 25 36 41 showed lesser retention rates than our study. The relatively lower retention rates observed in this study compared with many cited works may be attributed to the absence of rubber dam isolation, the school- and outreach-based setting with less-than-ideal clinical conditions, and the inherent challenges of applying sealants in young children. Furthermore, many of the studies reporting higher retention were conducted under controlled operatory conditions using different sealant materials, which may explain the discrepancy.

The prevention of caries development is primarily associated with effective sealant retention and the potential for fluoride release. 21 Additionally, the effectiveness of sealants in caries prevention may also be attributed to the residual material that remains within the depths of pits and fissures, offering continued protection even in cases of partial sealant loss. 14

The lower incidence of caries observed in the EWB group may be attributed to its higher retention rates and effective marginal seal. Conversely, a study by Mohanraj et al reported a higher caries incidence in the EWB group, suggesting that the increased filler content of the sealant may be a contributing factor. Filled RBSs tend to create rough surfaces and irregular margins, which can facilitate plaque accumulation and subsequently increase the risk of caries development. 36 38 The caries incidence seen at 12 months is higher in majority of the studies 8 12 13 14 15 16 17 21 36 40 41 ranging from 3.57 to 27.8%. The lower caries incidence observed at 12 months in this study compared with many cited works may be attributed to the continued protective effect of residual sealant, the favorable marginal adaptation of EWB, the relatively short follow-up duration, and differences in participant caries risk profiles.

Marginal discoloration increased over time, with a small percentage of restorations showing superficial or deep staining at the 12-month follow-up. At 6 months, no discoloration was seen in 95.2% children in one study 14 and another study 8 showed 100% of no discoloration cases. At 12 months, no discoloration was seen in 77.5% children in one study. 14 Although discoloration did not affect the functional integrity of the sealants, it may have aesthetic implications, particularly in anterior teeth.

Marginal adaptation remained excellent throughout the study period, with no detectable V-shaped defects in enamel or DEJ at any follow-up point. The sealants which show missing retention are taken as undetectable crevices along the margin. This finding suggests that the sealant material used had good adaptation properties, ensuring minimal microleakage and preventing secondary caries formation. In one study, after a 12-month follow-up, the absence of visible crevices along the periphery (Alpha rating) was observed 60.2% in EWB. This outcome may be attributed to the etching process, which can enhance the adhesion of sealant materials to enamel, thereby improving marginal adaptation. The presence of marginal gaps may result in marginal staining, often regarded as an early indicator of sealant failure. 40 At 6 months, no detectable crevice was seen ranging between 80 and 94.4% in four studies. 8 14 18 38 While at 12 months, no detectable crevice was seen in 60 and 83.3% in two studies. 14 18 The changes in marginal adaptation were minimal in both groups. The hydrophilic pit and fissure sealants demonstrated a sealing ability comparable to that of conventional hydrophobic sealants. This outcome may be attributed to their low viscosity and water sorption properties, as noted by Prabhakar et al's study. 6

The color match of restorations was initially perfect in all cases, but by 12 months, there was a noticeable decline. At 12 months, only 78.4% of restorations showed perfect shade matching, with 17.6% (Group I) and 19.6% (Group II) having clinically acceptable mismatches, and 4% (Group I) and 2% (Group II) showing aesthetically unacceptable mismatches. The sealants with full loss of retention were counted as no shade mismatch. At the 6th month, perfect shade match was seen in 95.2 and 100% children in two studies. 8 14 While at 12th month, only one study showed the color match parameter and perfect shade match in 77.5% children. 14 This decline in shade matching could be attributed to material wear, external staining, or minor surface degradation over time.

Surface smoothness was maintained well throughout the study. At 1 and 6 months, all restorations had a smooth surface comparable to adjacent tooth structures. At 12 months, a slight decrease was noted, with 2% of restorations exhibiting roughness, but none developing significant surface irregularities such as pits or fissures. The sealants with complete loss of the sealant were counted as restoration is as smooth as adjacent tooth structure. At the 6th month, the surface smoothness seen as smooth is seen in around 81% in one study 14 and 93% in another study. 8 While at the 12th month, the smooth surface is seen in 67.5% in only one study, 14 which is less than our study results. This indicates that despite minor changes, the sealants maintained good surface integrity over time.

This study used a split-mouth design to control for individual factors like diet and habits, enhancing validity by minimizing interpatient variability. A 12-month prospective approach allowed comprehensive assessment of sealant retention, caries development, and aesthetics using standardized clinical criteria. Comparisons between moisture-controlled and non-controlled groups provided key insights into sealant longevity. Statistical rigor was maintained through appropriate tests (Friedman and Mann-Whitney U), and blinding minimized evaluator bias.

This study had some limitations, including a 12-month follow-up that may not fully reflect the long-term effectiveness and retention loss of sealants. Clinical evaluation excluded tests like microleakage or bond strength, which could have offered deeper insights. Aesthetic assessments were based on subjective visual inspection, potentially affecting consistency, and rubber dam isolation was not used due to resource constraints. In addition, participant (child) blinding was not feasible, as the interventions were performed in different settings on separate days, which may have introduced potential bias. A further limitation of the study is the absence of explicit caries risk assessment in the inclusion criteria, which may limit the generalizability of the results.

This study evaluated only EWB under two clinical conditions; inclusion of additional sealant types could have provided broader comparative insights. Future research should explore such comparisons to strengthen the evidence base.

From a public health perspective, the findings carry significant implications. In many low- and middle-income settings, including India, large-scale oral health programs often rely on outreach and school-based delivery models where advanced isolation equipment and strict environmental control are not feasible. The demonstrated performance of EWB sealants under these real-world constraints highlights their practical utility in community dentistry. By ensuring effective caries prevention in challenging field conditions, such sealants can contribute to reducing the burden of untreated dental caries among school-aged children, offering a cost-effective and scalable preventive strategy.

## Conclusion

The study concludes that hydrophilic EWB sealants are effective in preventing occlusal caries, with comparable performance in both moisture-controlled and non-controlled conditions. Minor aesthetic changes did not impact functional outcomes. Their reliable retention under challenging conditions makes them ideal for large-scale pediatric and school-based oral health programs. In resource-limited settings such as India, where conventional moisture control is often difficult to achieve, their use represents a practical, cost-effective, and scalable strategy for caries prevention and improved oral health equity.

## Clinical Relevance Statement

Hydrophilic EWB sealants demonstrated effective caries prevention and comparable retention under both moisture-controlled and non-moisture-controlled conditions. Their reliable performance in less-than-ideal clinical environments supports their use in school-based and community oral health programs, particularly in resource-limited settings where conventional isolation methods are challenging.
